# Two new species of freshwater crabs of the genera *Eosamon* Yeo & Ng, 2007 and *Indochinamon* Yeo & Ng, 2007 (Crustacea, Brachyura, Potamidae) from southern Yunnan, China

**DOI:** 10.3897/zookeys.980.52186

**Published:** 2020-10-28

**Authors:** Zewei Zhang, Da Pan, Xiyang Hao, Hongying Sun

**Affiliations:** 1 Jiangsu Key Laboratory for Biodiversity and Biotechnology, College of Life Sciences, Nanjing Normal University, 1 Wenyuan Rd, Nanjing 210023, China Nanjing Normal University Nanjing China

**Keywords:** 16S rDNA, *Eosamon daiae* sp. nov., *Indochinamon malipoense* sp. nov., new species, Potamidae, Potamiscinae, taxonomy

## Abstract

Two new species of potamid crabs, *Eosamon daiae***sp. nov.** and *Indochinamon malipoense***sp. nov.** are described from the Sino-Burmese border, southwestern Yunnan and from the Sino-Vietnamese border, southeastern Yunnan, China. The two new species can be distinguished from their closest congeners by several characters, among which is the form of the first gonopod structures. Molecular analyses based on partial mitochondrial 16S rDNA sequences also support the systematic status of these new taxa.

## Introduction

China has the most freshwater crab species in the world and Yunnan is the epicenter of this diversity, with over 60 species in 17 genera (Dai 1999; Chu et al. 2018a, b; Naruse et al. 2018). Despite this, the biodiversity of freshwater crabs in this region appears to be still underestimated, especially in the remote areas (Chu et al. 2018b). In this paper we describe two new species belonging to two genera, *Eosamon* Yeo & Ng, 2007, and *Indochinamon* Yeo & Ng, 2007, from the Sino-Burmese and Sino-Vietnamese border areas in Yunnan Province, China. *Eosamon* and *Indochinamon* are widely distributed in the Indochina Peninsula (Yeo and Ng 2007). Including the two new species described in the present study, *Eosamon* and *Indochinamon* respectively contain 12 and 40 species (Yeo and Ng 2007; Yeo 2010; Naruse et al. 2011, 2018; Van et al. 2016; Ng and Mar 2018).

## Material and methods

**Figure 1.**
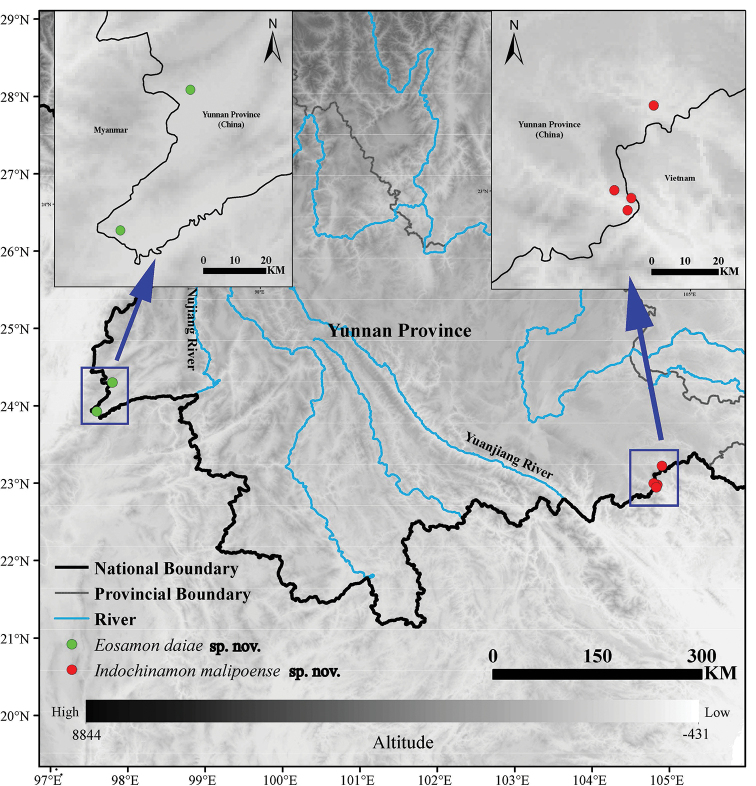
Locality of sampling sites for *Eosamon daiae* sp. nov. and *Indochinamon malipoense* sp. nov. in southwestern and southeastern Yunnan Province, China.

Specimens were collected from southwestern and southeastern Yunnan (Fig. 1), preserved in 95% ethanol and identified via a stereo dissection microscope (Nikon SMZ645). Materials examined are deposited in the Jiangsu Key Laboratory for Biodiversity and Biotechnology, College of Life Sciences, Nanjing Normal University (NNU), Nanjing, China. Carapace width and length were measured in millimeters. The terminology used here follows Guinot et al. (2013). The following abbreviations are used: G1 for male first gonopod, G2 for male second gonopod, a.s.l. for above sea level.

**Molecular data.** Genomic DNA was extracted from gill tissue using the the Trelief^TM^ Animal Genomic DNA kit (Tsingke). 16S rDNA sequence was selected for amplification with polymerase chain reaction (PCR) using the primers 1471 and 1472 (Crandall and Fitzpatrick 1996). Parameters for PCR were as follows: initial denaturation at 95 °C for 3 min, followed by 35 cycles of 15 sec at 95 °C, 15 sec at 48 °C, 45 sec at 72 °C, and a subsequent 7 min final extension step at 72 °C. Both ends of PCR products were then sequenced using an ABI 3730 automatic sequencer. Sequences were assembled using SEQMAN II 5.05. Sequences of different haplotypes have been deposited in the Genbank (accession numbers listed in Table 1). To confirm the systematic position of newly described taxa, a total of 64 sequences were used in phylogenetic analyses, including 56 downloaded sequences (Table 1).

**Table 1.** 16S rDNA sequences sampled in this study.

Species	Accession No.	Voucher No.	Reference
*Amamiku amamensis*	AB428457	–	Shih et al. 2009
*Aparapotamon grahami*	AB428489	–	Shih et al. 2009
*Apotamonautes hainanensis*	AB428459	–	Shih et al. 2009
*Arquatopotamon jizushanense*	KY963596	–	Chu et al. 2017
*Artopotamon latopeos*	MH045061	–	Chu et al. 2018a
*Beccumon jarujini*	AB428479	–	Shih et al. 2009
*Candidiopotamon rathbunae*	AB208598	–	Shih et al. 2006
*Chinapotamon glabrum*	AB428451	–	Shih et al. 2009
*Demanietta renongensis*	AB428475	–	Shih et al. 2009
*Diyutamon cereum*	LC198519	–	Huang et al. 2016
*Eosamon boonyaratae*	AB428487	–	Shih et al. 2009
*Eosamon daiae* sp. nov.	MT887282	NNU 190508	This study
*Eosamon daiae* sp. nov.	MT887283	NNU 190509	This study
	MT887280	NNU 190405	This study
	MT887281	NNU 190406	This study
*Eosamon lushuiense*	MT887284	NNU LSWG1503	This study
*Eosamon smithianum*	AB428486	–	Shih et al. 2009
*Eosamon tengchongense*	MT887285	NNU TCML02	This study
*Eosamon yotdomense*	AB428485	–	Shih et al. 2009
*Esanpotamon namsom*	AB428463	–	Shih et al. 2009
*Flabellamon* sp.	AB428472	–	Shih et al. 2009
*Geothelphusa albogilva*	AB127366	–	Shih et al. 2004
*Geothelphusa fulva*	AB428456	–	Shih et al. 2009
*Geothelphusa olea*	AB428455	–	Shih et al. 2009
*Hainanpotamon fuchengense*	AB428461	–	Shih et al. 2009
*Himalayapotamon atkinsonianum*	AB428510	–	Shih et al. 2009
*Huananpotamon angulatum*	AB428454	–	Shih et al. 2009
*Indochinamon malipoense* sp. nov.	MT887278	NNU 180601	This study
	MT887279	NNU 180602	This study
*Indochinamon ou*	AB428481	–	Shih et al. 2009
*Indochinamon tannanti*	AB428482	–	Shih et al. 2009
*Johora johorensis*	AB290620	–	Yeo et al. 2007
*Johora murphyi*	AB290621	–	Yeo et al. 2007
*Kanpotamon duangkhaei*	AB428471	–	Shih et al. 2009
*Kukrimon cucphuongensis*	AB428483	–	Shih et al. 2009
*Megacephalomon kittikooni*	AB428462	–	Shih et al. 2009
*Mindoron balssi*	AB428464	–	Shih et al. 2009
*Minpotamon nasicum*	AB428450	–	Shih et al. 2009
*Minutomon shanweiense*	LC176065	–	Huang et al. 2016
*Nanhaipotamon formosanum*	AB212867	–	Shih et al. 2005
*Nanhaipotamon nanriense*	AB212868	–	Shih et al. 2005
*Neotiwaripotamon jianfengense*	AB428460	–	Shih et al. 2009
*Ovitamon artifrons*	AB428466	–	Shih et al. 2009
*Parapotamon spinescens*	AB428467	–	Shih et al. 2009
*Pararanguna semilunata*	AB428490	–	Shih et al. 2009
*Paratelphusula gibbosa*	AB428512	–	Shih et al. 2009
*Potamiscus loshingense*	AB428488	–	Shih et al. 2009
*Potamiscus yiwuensis*	AB428476	–	Shih et al. 2009
*Potamiscus yunnanense*	AB290629	–	Yeo et al. 2007
*Potamon fluviatile*	AB428514	–	Shih et al. 2009
*Pudaengon sakonnakorn*	AB428484	–	Shih et al. 2009
*Pupamon nayung*	AB428477	–	Shih et al. 2009
*Ryukyum yaeyamense*	AB428458	–	Shih et al. 2009
*Semicirculara lincangense*	MH045059	–	Chu et al. 2018a
*Shanphusa curtobates*	AB428478	–	Shih et al. 2009
*Sinolapotamon anacoluthon*	AB428453	–	Shih et al. 2009
*Socotrapotamon nojidense*	AB428493	–	Shih et al. 2009
*Tenuilapotamon latilum*	AB428468	–	Shih et al. 2009
*Tenuipotamon huaningense*	AB428491	–	Shih et al. 2009
*Thaiphusa* sp.	AB428474	–	Shih et al. 2009
*Tomaculamon pygmaeus*	AB428473	–	Shih et al. 2009
*Trichopotamon daliense*	AB428492	–	Shih et al. 2009
*Yarepotamon gracilipa*	AB428452	–	Shih et al. 2009
*Yuebeipotamon calciatile*	LC176064	–	Huang et al. 2016

**Phylogenetic analyses.** Sequences were aligned using MAFFT 7.310 (Katoh and Standley 2013) based on the G-INS-I method. Gapped positions were treated as missing data. Maximum likelihood (ML) analysis for the dataset was performed using IQ-TREE 1.6.12 (Nguyen et al. 2015). The best substitution model was determined by ModelFinder (Kalyaanamoorthy et al. 2017). Node reliability was obtained through 1000 ultrafast bootstrap replicates (Minh et al. 2013). For Bayesian inference (BI), the best-fitting model was determined by MrModeltest 2.4 (Nylander 2004), selected by the Akaike information criterion (AIC). The best model obtained was GTR+I+G. Bayesian inference was performed using MRBAYES 3.2.6 (Ronquist et al. 2012) with four chains for 20 million generations, with trees sampled every 5000 generations. The first 25% of MCMC chains were discarded as burn-in. The sampled parameters and convergence of four MCMC chains were investigated using TRACER 1.6 (Rambaut et al. 2014). The effective sampling sizes for all parameters were more than 200. Bootstrap support (BS) and Bayesian posterior probability (BPP) were used to assess statistical support.

## Results

### Taxonomy

#### Family Potamidae Ortmann, 1896

**Subfamily Potamiscinae Bott, 1970**

**Genus *Eosamon* Yeo & Ng, 2007**

##### Eosamon daiae

Taxon classificationPlantaeDecapodaPotamidae

Zhang & Sun sp. nov.

FA288484-0745-5688-BE82-236F51922D6D

http://zoobank.org/3753C63F-4E88-4650-AC9D-D21A2A8880B7

**Figure 2.**
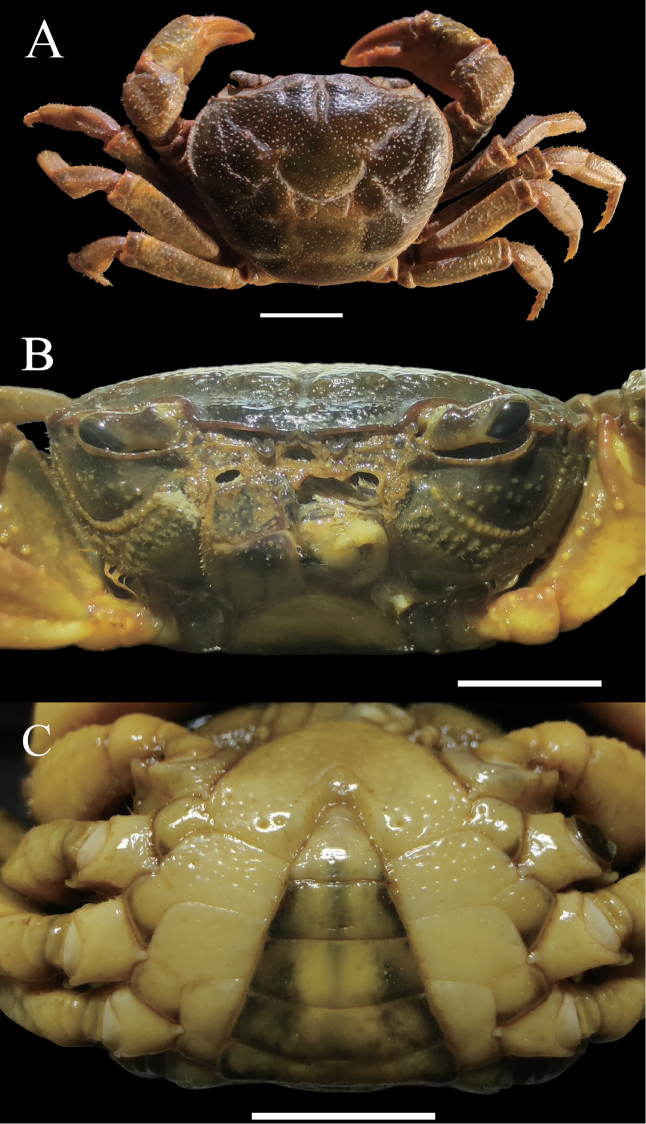
*Eosamon daiae* sp. nov. holotype, male, 26.6 × 22.2 mm, NNU 190503 **A** dorsal view **B** frontal view of cephalothorax **C** ventral view showing anterior thoracic sternum and pleon. Scale bars: 1.0 cm.

**Figure 3.**
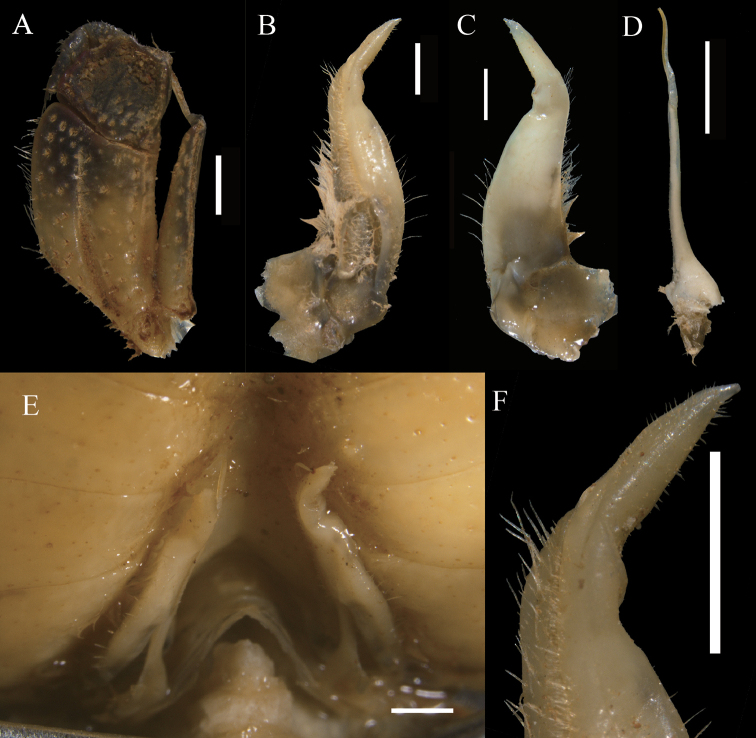
*Eosamon daiae* sp. nov. holotype, male, 26.6 × 22.2 mm, NNU 190503 **A** left third maxilliped **B** left G1 (ventral view) **C** left G1 (dorsal view) **D** left G2 **E** sterno-abdominal cavity with G1 *in situ***F** G1 terminal segment (ventral view). Scale bars: 1.0 mm.

**Figure 4.**
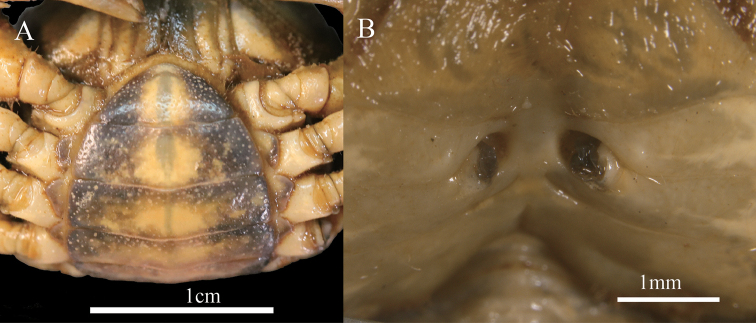
*Eosamon daiae* sp. nov. paratype, female, 20.1 × 16.5 mm, NNU 190505 **A** abdomen **B** vulvae. Scale bars: 1.0 cm (**A**); 1.0 mm (**B**).

**Figure 5.**
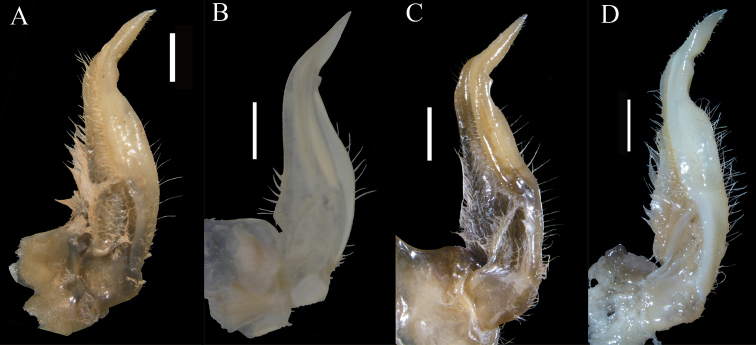
The ventral view of left G1 **A***Eosamon daiae* sp. nov. holotype, male, 26.6 × 22.2 mm, NNU 190503 **B***Eosamon tumidum*, male, 23.2 × 18.7 mm, IZCAS CB11382 **C***Eosamon lushuiense*, male, 23.7 × 19.9 mm, NNU 162821 **D***Eosamon tengchongense*, male, 37.9 × 30.1 mm, NNU 193261. Scale bars: 1.0 mm.

**Figure 6.**
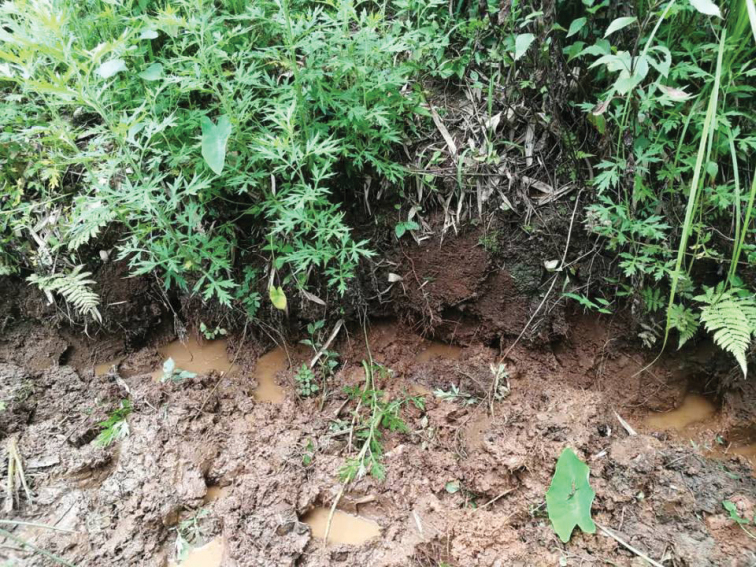
Habitat of *Eosamon daiae* sp. nov., the moist mud burrows at the type locality, Tianbao Town, Yunnan Province, China.

Figs 2 , 3 , 4 , 5 , 6

###### Material examined.

***Holotype***: China • 1 male, 26.6 × 22.2 mm, NNU 190503; Yunnan Province, Dehong Prefecture, Longchuan County, Longba Town, Bangyang Village; 24°18'15"N, 97°47'56"E; 998 m a.s.l.; 5 May 2019; leg. Xiyang Hao & Zewei Zhang. ***Paratypes***: China • 1 female, 20.1 × 16.5 mm, NNU 190505; same data as holotype • 1 male, 24.8 × 20.4 mm, NNU 190504; same data as holotype. Other material: China • 3 males, 20.9 × 17.5 mm, NNU 190401, 23.0 × 19.3 mm, NNU 190402, 21.5 × 17.8 mm, NNU 190403; same data as holotype. CHINA • 1 female, 19.7 × 16.5 mm, NNU 190407; Yunnan Province, Ruili City, Nongdao Town, Dengga Village; 23°55'51"N, 97°47'56"E; 887 m a.s.l.; 4 May 2019, leg. Xiyang Hao & Zewei Zhang.

###### Comparative material.

*Eosamon tumidum* (Wood-Mason, 1871): China • 1 male, 23.2 × 18.7 mm, IZCAS CB11382; Yunnan Province, Sipaishan; 1964; *Eosamon tengchongense* (Dai & Chen, 1985): China • 1 male, 37.9 × 30.1 mm, NNU 193261; Yunnan Province, Lianghe County; 9 May 2019; leg. Xiyang Hao & Zewei Zhang; *Eosamon lushuiense* (Dai & Chen, 1985): China • 1 male, 23.7 × 19.9 mm, NNU 162821; Yunnan Province, Lushui City; 4 May 2016; leg. Kelin Chu, Pengfei Wang & Hongying Sun.

###### Diagnosis.

Carapace slightly broader than long, dorsal surface strongly convex, densely pitted (Fig. 2A). Third maxilliped exopod reaching proximal 1/3 of merus length, with long flagellum (Fig. 3A). Male pleon triangular, lateral margin almost straight (Fig. 2C), G1 subterminal segment broad, terminal segment relatively short, clearly sinuous, inferior margin of terminal segment straighter than superior margin, tip of terminal segment gradually tapering to a sharp tip (Fig. 3F), subterminal segment about 3.3 times as long as terminal segment (Fig. 3B, C). G1 strongly curved outwards, not reaching pleonal locking mechanism *in situ* (Fig. 3E). Female pleon ovate (Fig. 4A), vulvae on suture between thoracic sternites 5/6, ovate, opening inner upwards, vulvar cover margin slightly arched (Fig. 4B).

###### Description.

Carapace about 1.2 times broader than long (*N* = 6), subquadrate, dorsal surface strongly convex transversely and longitudinally, punctate, smooth, regions distinctly defined (Fig. 2A); anterolateral region lined with granules; posterolateral margin with rugae (Fig. 2A); cervical groove and H-shaped groove between gastric and cardiac regions deep, distinct (Fig. 2A). Epigastric region distinct, separated by narrow groove (Fig. 2A). Postfrontal lobe slightly convex, separated medially by Y-shaped groove extending to frontal region (Fig. 2A). Front deflexed downwards, postorbital region distinctly concave (Fig. 2A, B). Dorsal orbital margin ridged, external orbital angle triangular, epibranchial tooth pointed, clearly demarcated from external orbital tooth by gap; supraorbital and infraorbital margins cristate (Fig. 2A, B). Branchial regions relatively flat, smooth with dense dots (Fig. 2A). Pterygostomial regions smooth with several granules; epistome lateral margins sinuous; median lobe triangular (Fig. 2B).

Third maxilliped merus about 1.2 times as broad as long, trapezoidal, with median depression; ischium about 1.2 times as long as broad, rectangular, with distinct median sulcus; exopod reaching proximal 1/3 of merus length with flagellum (Fig. 3A).

Chelipeds slightly unequal; merus trigonal in cross section, margins crenulated (Fig. 2A); carpus with sharp spine on inner-distal angle, with spinule at base and striae (Fig. 2A); manus of major chela with convex granules, about 1.5 times as long as high (Fig. 2A); dactylus bent inwards (Fig. 2A), gap narrow when fingers closed, cutting edge lined with irregular sized teeth (Fig. 2A).

Ambulatory legs relatively stout, dactylus slender with spine-like setae (Fig. 2A); second ambulatory leg merus about 1.3 times as long as dactylus; last leg with propodus about 1.7 times as long as broad, slightly shorter than dactylus (Fig. 2A).

Male thoracic sternum generally smooth and pitted; sternites 3, 4 fused without median suture (Fig. 2C). Female thoracic sternum wider, sutures the same as male.

Male pleon triangular, third somite widest; sixth somite about 2.2 times broader than long; telson triangular, with about 1.3 times as broad as long; the lateral margin of pleon almost straight (Fig. 2C); sterno-pleonal cavity reaching anteriorly to level of mid-length of cheliped coxae bases, broad, deep, median longitudinal groove between sternites 7, 8 long (Fig. 3E). Female pleon ovate, surface pitted; sixth somite about 2.8 times as broad as long; telson semicircular, terminal gently protuberant, about 2.3 times as broad as long (Fig. 4A).

G1 stout, tip of terminal segment not reaching pleonal locking mechanism *in situ* (Fig. 3E); subterminal segment stout, about 3.3 times as long as terminal segment (Fig. 3B, C); G1 terminal segment cone-shape, bent outwards, inferior margin of terminal segment straighter than superior margin, tip of G1 terminal segment gradually tapering to sharp tip (Fig. 3F). G2 slightly longer than G1, basal segment about 2.1 times as long as distal segment (Fig. 3D). Female vulvae on suture between thoracic sternites 5/6, ovate, opening inwards towards the median of the cavity, vulvar cover slightly arched (Fig. 4B).

###### Live coloration.

Carapace is usually dark brown, while chelipeds and ambulatory legs are usually light brown in life.

###### Etymology.

The species is named after the late Prof. Aiyun Dai, who made a huge contribution to freshwater crab studies in China during her lifetime.

###### Remarks.

*Eosamon daiae* sp. nov. can be distinguished from other *Eosamon* species by the combination of male abdomen with straight lateral margins, relatively broad G1 subterminal segment, conical and straight G1 terminal segment, the superior margin of G1 terminal segment curved and the inferior margin of G1 terminal segment comparatively straight.

*Eosamon daiae* sp. nov. is morphologically and geographically closest to *E. tumidum* (Wood-Mason, 1871), *E. tengchongense* (Dai & Chen, 1985) and *E. lushuiense* (Dai & Chen, 1985). These species are characterized by a male abdomen with straight lateral margins and superficially similar G1 structure (Fig. 5). But *Eosamon daiae* sp. nov. can be distinguished by the fact that the superior margin of G1 terminal segment is curved and the inferior margin is comparatively straight (Fig. 3F, 5A) (versus superior margin comparatively straight and inferior margin slightly curved in both *E. tumidum* and *E. lushuiense*, Fig. 5B, C; outer and inner margins all comparatively curved in *E. tengchongense*, Fig. 5D); the distal part of G1 subterminal segment slightly sunken (Fig. 5A) (versus barely sunken in *E. tumidum*, Fig. 5B, prominently sunken in *E. tengchongense*, Fig. 5D). Other characters as shown in Table 2.

###### Distribution and habitat.

*Eosamon daiae* sp. nov. was found in Bangyang Village (24°18'15"N, 97°47'56"E, 998 m a.s.l.), Longba Town, Longchuan County and Dengga Village (23°55'51"N, 97°47'56"E, 887 m a.s.l.), Nongdao Town, Ruili City, Dehong Prefecture in the frontier of Yunnan, China (Fig. 1). They reside in moist mud burrows on the ridge of field and under low bushes (Fig. 6).

The new species was found not distant from localities with *E. tengchongense*. *Indochinamon* dominates the areas surrounding the new species, with *I. edwardsi*, *I. andersonianum*, *I. boshanense* and *I. gengmaense* having been recorded.

**Table 2.** Morphological differences for *Eosamon daiae* sp. nov., *Eosamon tumidum*, *Eosamon lushuiense* and *Eosamon tengchongense*.

Character	*E. daiae* sp. nov.	*E. tumidum* (cf. Dai 1999: pl. 174 fig. 91)	*E. lushuiense* (cf. Dai 1999: pl. 175 fig. 92)	*E. tengchongense* (cf. Dai 1999: pl. 177 fig. 93)
Carapace	Strongly convex (Fig. 2A, B)	Slightly convex	Slightly convex	Slightly convex
Margins of G1 terminal segment	superior margin Curved, inferior margin comparatively straight (Fig. 5A)	superior margin comparatively straight, inferior margin slightly curved (Fig. 5B)	superior margin comparatively straight, inferior margin slightly curved (Fig. 5C)	superior margin and inferior margin, comparatively curved (Fig. 5D)
Distal part of G1 subterminal segment	slightly sunken (Fig. 5A)	barely sunken (Fig. 5B)	slightly sunken (Fig. 5C)	obviously sunken (Fig. 5D)
Ratio of G1 subterminal segment to terminal segment	3–3.3	3.2	2.9	3.1

#### Genus *Indochinamon* Yeo & Ng, 2007

##### Indochinamon malipoense

Taxon classificationPlantaeDecapodaPotamidae

Zhang & Sun sp. nov.

0704422B-76C0-5A56-9390-D2F05DC40DA9

http://zoobank.org/6B741968-8048-454C-8040-50D3BC581A5F

Figs 7 , 8 , 9 , 10

###### Material examined.

***Holotype***: China • 1 male, 53.0 × 42.7 mm, NNU 180505; Yunnan Province, Wenshan Prefecture, Malipo County, Tianbao Town, Bajiaoping Village; 22°58'53"N, 104°50'27"E; 1075 m a.s.l.; 5 April 2018; leg. Zhan Zhang, Zewei Zhang & Hongying Sun. ***Paratypes***: China • 1 female, 48.0 × 38.2 mm, NNU 180603; Yunnan Province, Wenshan Prefecture, Malipo County, Babu Town; 23°13'29"N, 104°54'04"E; 550 m a.s.l.; 6 April 2018, leg. Zhan Zhang, Zewei Zhang & Hongying Sun • 2 males, 63.2 × 49.0 mm, NNU 180501, 60.5 × 48.0 mm, NNU 180506, same data as holotype.

###### Comparative material.

*Indochinamon changpoense* Dai, 1995: China • 1 male, 44.1 × 35.6 mm, NNU 161701; Yunnan Province, Jinping County Changpotou; 17 May 2016; leg. Kelin Chu, Pengfei Wang & Hongying Sun; *Indochinamon tannanti* Rathbun, 1904: China • 1 male, 43.3 × 34.9 mm, NNU 180801; Yunnan Province, Hekou County; 8 April 2018; leg. Zhan Zhang, Zewei Zhang & Hongying Sun.

###### Diagnosis.

**Figure 7.**
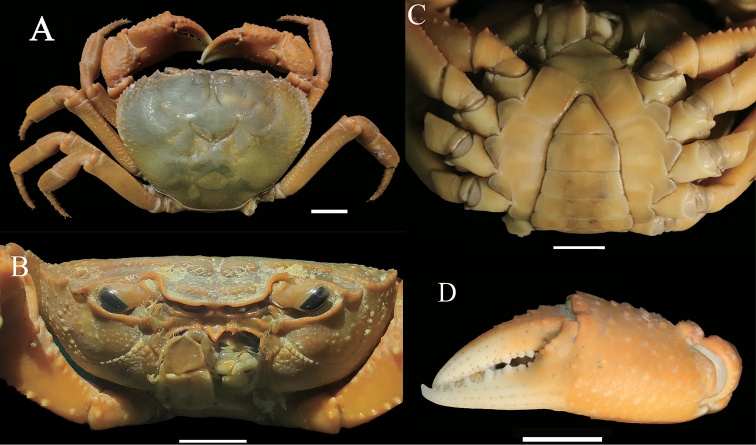
*Indochinamon malipoense* sp. nov. holotype, male, 53.0 × 42.7 mm, NNU 180505 **A** dorsal view **B** frontal view of cephalothorax **C** ventral view showing anterior thoracic sternum and pleon **D** outer surfaces of left major chela. Scale bars: 1.0 cm.

**Figure 8.**
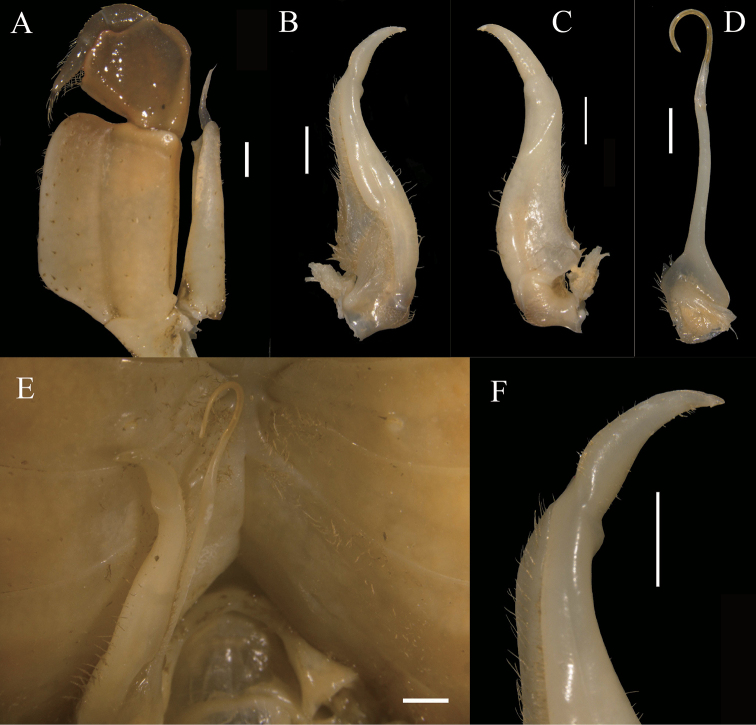
*Indochinamon malipoense* sp. nov. holotype, male, 53.0 × 42.7 mm, NNU 180505 **A** left third maxilliped **B** left G1 (ventral view) **C** left G1 (dorsal view) **D** left G2 **E** sterno-pleonal cavity with right G1 *in situ***F** left G1 terminal segment (ventral view). Scale bars: 1.0 mm.

**Figure 9.**
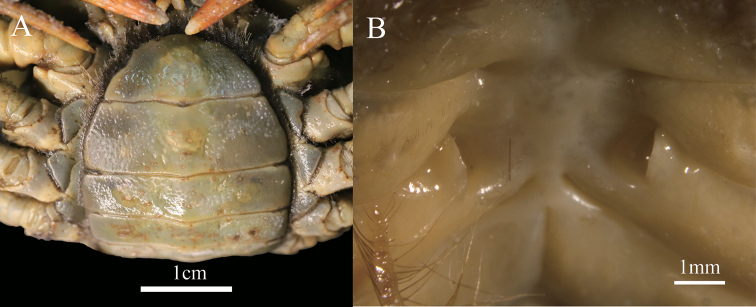
*Indochinamon malipoense* sp. nov. paratype, female, 48.0 × 38.2 mm, NNU 180603 **A** abdomen **B** vulvae.

Carapace broader than long, dorsal surface glabrous, gently convex; regions indistinctly defined; anterolateral margin lined with obvious granules (Fig. 7A). Third maxilliped exopod with flagellum (Fig. 8A). Male pleon triangular, lateral margin of sixth somite distinctly convex; telson triangular, tip rounded (Fig. 7C); G1 terminal segment distinctly curved, subterminal segment about 3.2 times as long as terminal segment (Fig. 8B, C); G1 strongly curved outwards, not reaching pleonal locking mechanism *in situ* (Fig. 8E). Female pleon ovate (Fig. 9A), vulvae on thoracic sternite 6, subrotund, opening inner, ventrolateral margin arched distinctly (Fig. 9B).

###### Description.

Carapace about 1.2 – 1.3 times broader than long (*N* = 4), subtrapezoidal, dorsal surface gently convex, glabrous; anterolateral region lined with granules, border with spinose granulation (Fig. 7A); cervical groove shallow, inconspicuous; H-shaped groove between gastric and cardiac regions shallow but distinct (Fig. 7A). Front slightly deflexed, with anterior border emarginated medially (Fig. 7A, B); postfrontal lobe distinctly convex, separated medially by Y-shaped groove; postorbital cristae obviously convex, separated from postfrontal lobe by distinct groove (Fig. 7A); postorbital region distinctly concave (Fig. 7A, B). Posterolateral margin comparatively smooth with few rugae; branchial regions relatively flat, smooth (Fig. 7A). External orbital angle acutely triangular; epibranchial tooth with sharp protuberance, separated from external orbital angle by distinct cleft (Fig. 7A). supraorbital, infraorbital margins cristate; pterygostomial regions comparatively smooth with several granules (Fig. 7B). Epistome superior margin cristate, inferior margin slightly curved with median triangle (Fig. 7B).

Ischium of third maxilliped elongate rectangular, about 1.3 times longer than broad, with distinct, longitudinal median sulcus; merus trapezoidal, about 1.1 times broader than long; exopod reaching beyond base of merus slightly, with short flagellum, about half the width of the merus (Fig. 8A).

Chelipeds unequal (Fig. 7A); merus margins crenulated (Fig. 7C); carpus with sharp spine at inner-distal angle, spinules and granules at base (Fig. 7A); outer surface of manus with convex granules, about 1.3 times as long as high; immovable, movable fingers curved inwards, with irregular teeth; gape narrow when fingers closed (Fig. 7D).

Ambulatory legs relatively slender, dactylus slender, with spine-like setae (Fig. 7A); second ambulatory leg merus about 1.8 times as long as dactylus; last leg with propodus about 2.7 times as long as broad, slightly shorter than dactylus (Fig. 7A).

Thoracic sternum glabrous, sternites 1, 2 completely fused to form triangular structure; suture between sternites 2, 3 distinct (Fig. 7C); suture between sternites 3, 4 shallow (Fig. 7C); sterno-pleonal cavity reaching anteriorly to level of mid-length of cheliped coxae bases, median longitudinal groove between sternites 7, 8 long (Fig. 8E). Male pleon triangular, third somite widest; sixth somite width 2.0 times length; telson triangular, width 1.4 times length, tip of telson round (Fig. 7C). Female pleon ovate, smooth, pitted; sixth somite about 2.9 times as broad as long, telson semicircular, about 2.2 times as broad as long (Fig. 9A).

G1 stout, bent; tip of terminal segment not reaching pleonal locking mechanism *in situ* (Fig. 8E); subterminal segment stout, about 3.2 times as long as terminal segment (Fig. 8B, C); terminal segment slender, unciform, clearly curved outwards, inferior and superior margins curved (Fig. 8E, F); base of G1 terminal segment slightly inflated, distal part tapered (Fig. 8F); G2 distinctly longer than G1, subterminal segment about 1.2 times as long as terminal segment (Fig. 8D). Female vulvae on thoracic sternite 6, ovate, opening inwards towards median of cavity, vulvar cover margin slightly arched (Fig. 9B).

###### Live coloration

. The crabs usually have two colors: brownish-red (Fig. 11A) and yellowish-cyan (Fig. 11B). From the type locality, Tianbao Town, both brownish-red and yellowish-cyan crabs have been found, while from Babu Town, only yellowish-cyan crabs have been found. Morphologically, there is no distinct difference between individuals with different colors. Similar color variation also can be seen in another potamid crab, *Geothelphusa pingtung* Tan & Liu, 1998 (Shy et al. 2019).

**Figure 10.**
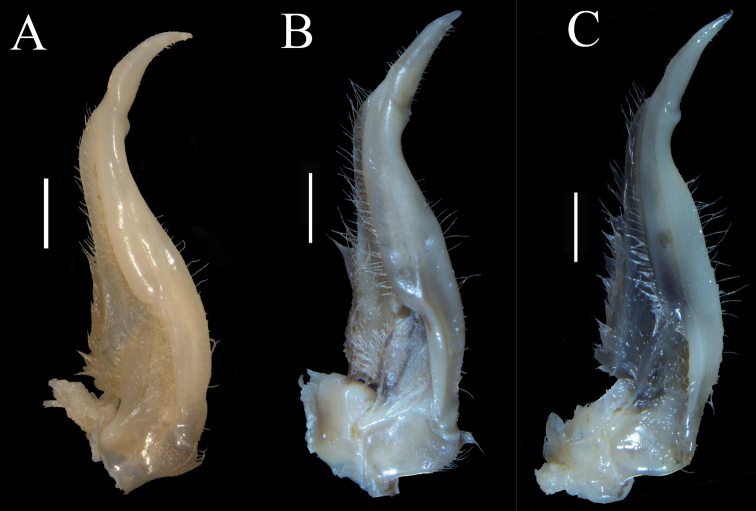
The ventral view of left G1 **A***Indochinamon malipoense* sp. nov. holotype, male, 53.0 × 42.7 mm, NNU 180505 **B***Indochinamon tannanti* male, 43.3 × 34.9 mm, NNU 180801 **C***Indochinamon changpoense* male, 44.1 × 35.6 mm, NNU 161701. Scale bars: 1.0 mm.

**Figure 11.**
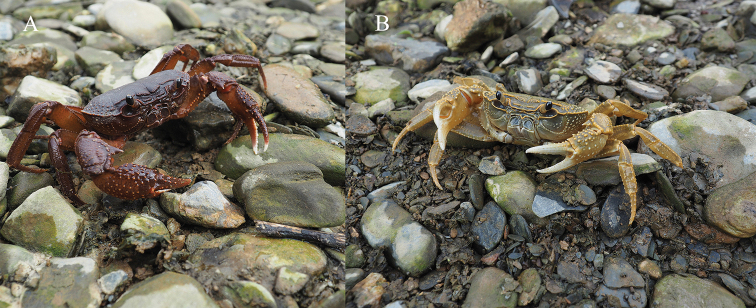
Color in life of *Indochinamon malipoense* sp. nov. **A** brownish-red male **B** yellowish-cyan male. Photographs by Hongying Sun, 5 April 2018, Tianbao Town, Yunnan Province, China.

###### Etymology.

This species is named after the type locality, Malipo County, Yunnan Province, China.

###### Remarks.

Based on the morphology of G1, Ng and Mar (2018) separated *Indochinamon* into several groups. The G1 terminal segment of *I. malipoense* sp. nov. is similar to a large group including the type species, *I. villosum* (Yeo & Ng, 1998). Within this group, *I. malipoense* sp. nov. closely resembles *I. ahkense* Naruse, Chia & Zhou, 2018, *I. bavi* Naruse, Nguyen & Yeo, 2011, *I. changpoense* (Dai, 1995), *I. daweishanense* (Dai, 1995), *I. kimboiense* Naruse, Nguyen & Yeo, 2011, *I. orleanis* (Rathbun, 1904), *I. ou* (Yeo & Ng, 1998), *I. parpidum* Naruse, Chia & Zhou, 2018, *I. tannanti* (Rathbun, 1904) and *I. yunlongense* (Dai, 1995), as their G1s are gently bent and G1 terminal segments are relatively slender and elongate (cf. Yeo and Ng 1998; Dai 1999; Naruse et al. 2011, 2018; Ng and Mar 2018). But *I. malipoense* sp. nov. can be distinguished from other species by the obviously curved G1 terminal segment.

All *Indochinamon* species have a well-developed flagellum on the exopod of the third maxilliped. The length of the flagellum varies among species. In some species, the flagellum does not exceed the width of the merus, e.g., *I. tannanti*, *I. changpoense*, *I. gengmaense* (Dai, 1995), *I. guttus* (Yeo & Ng, 1998), *I. hispidum* (Wood-Mason, 1871), *I. jinpingense*, *I. mieni* (Dang, 1967) and *I. yunlongense*. In *I. malipoense* sp. nov., the flagellum is about half the width of the merus, which is shorter than that in other species.

The G1 of *I. malipoense* sp. nov. is very similar to *I. tannanti*, *I. changpoense*, *I. ahkense*, and *I. daweishanense*. They are also geographically close. But *I. malipoense* sp. nov. can be distinguished from the similar *I. tannanti* and *I. changpoense* by several characters (Table 3), notably, the carapace regions are indistinctly defined (Fig. 7A) (versus distinctly defined in *I. tannanti* and *I. changpoense* (Dai 1999)), the G1 terminal segment is obviously curved, unciform (Fig. 10A) (versus slightly curved, conical in both *I. tannanti* and *I. changpoense*, Fig. 10B, C)), the base of the G1 terminal segment is slightly inflated (Fig. 8F) (versus nearly straight in both *I. tannanti* and *I. changpoense*, Fig. 10B, C). The G1 structure of *I. malipoense* sp. nov. is also similar to *I. ahkense* (Naruse et al. 2018: fig. 4) and *I. daweishanense* (Dai 1999: fig. 87) by relatively slender terminal segment. However, the G1 terminal segment is more curved in *I. malipoense* sp. nov. and stronger bent outward in *I. daweishanense*. The carapace of *I. malipoense* sp. nov. is superficially similar to *I. ahkense* by smooth and shallow grooves of the dorsal surface. In *I. ahkense*, the carapace is subquadrate (versus subtrapezoidal in *I. malipoense* sp. nov.) and flatter (versus slightly convex in *I. malipoense* sp. nov.).

In *I. khinpyae*, the carapace and G1 show considerable variations (Ng and Mar 2018). In smaller individuals, the carapace is less sculptured and the G1 terminal segment is shorter and straighter (Ng and Mar 2018). In *I. malipoense* sp. nov., the morphology of the carapace is relatively stable while the ratio of G1 subterminal segment to terminal segment varies in sampled individuals.

**Table 3.** Morphological differences for *Indochinamon malipoense* sp. nov., *Indochinamon tannanti* and *Indochinamon changpoense*.

Character	*I. malipoense* sp. nov.	*I. tannanti* (cf. Dai 1999: pl. 161 fig. 83)	*I. changpoense* (cf. Dai 1999: pl. 164 fig. 85)
carapace	gently convex, regions indistinctly defined (Fig. 7A)	flat, regions distinctly defined	gently convex, regions distinctly defined
G1 terminal segment	obviously curved, unciform (Fig. 10A)	slightly curved, conical, with short, conspicious setae,tip tapering (Fig. 10B)	slightly curved, conical, with few very short setae, dorsal lobe of pleopod opening visible (Fig. 10C)
base of G1 terminal segment	slightly inflated (Fig. 10A)	nearly straight (Fig. 10B)	nearly straight (Fig. 10C)
Ratio of G1 subterminal segment to terminal segment	2.8–3.2	2.7	2.9

###### Distribution and habitat.

*Indochinamon malipoense* sp. nov. was collected from Tianbao Town (22°58'53"N, 104°50'27"E, 1075 m a.s.l.; 22°56'58"N, 104°49'48"E, 223 m a.s.l.; 23°00'07"N, 104°47'42"E, 979 m a.s.l.) and Babu Town (23°13'29"N, 104°54'04"E, 550 m a.s.l.) located in the frontier between China and Vietnam, Malipo County, Wenshan Prefecture in Yunnan, China. They were found under rocks in mountain streams with altitudes of 200–1100 m.

*Indochinamon ahkense*, *I. changpoense*, *I. daweishanense*, *I. jinpingense*, *I. tannanti* and *Somanniathelphusa brevipodum* Tai, Song, He, Cao, Xu & Zhong, 1975, have been recorded near the distribution areas of *I. malipoense* sp. nov..

## Molecular results

**Figure 12.**
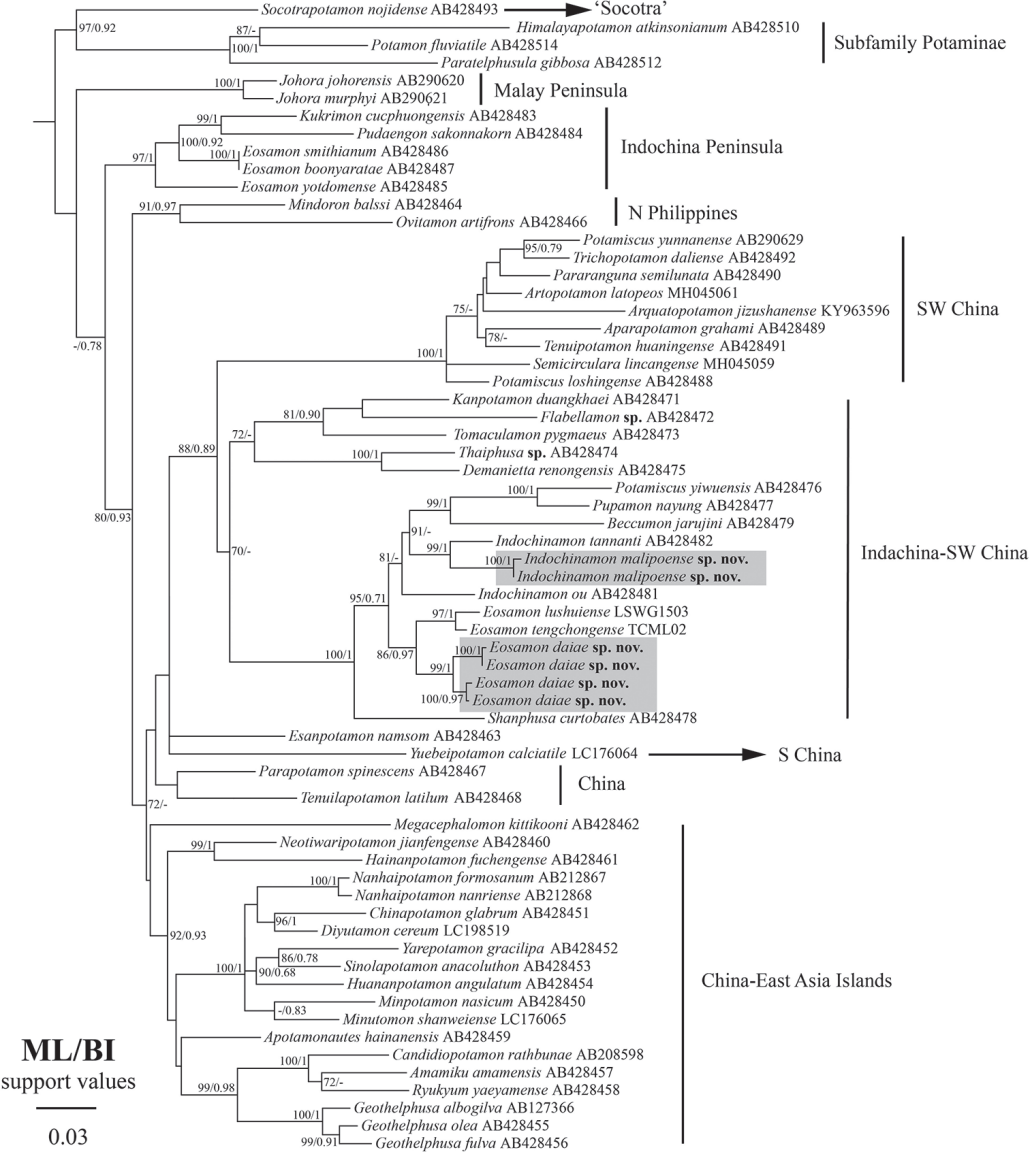
Phylogenetic tree reconstructed based on partial mitochondrial 16S rDNA sequences. The two new species are colored gray. Values at the nodes represent bootstrap (BS) values and posterior probability (BPP) values for ML and BI, respectively. Support values over 70/0.7 (BS/BPP) are provided.

In the present phylogenetic analyses, 60 species from 48 genera were included (Table 1). Phylogenetic trees reconstructed using BI and ML resulted in similar topologies. The phylogenetic trees indicate that two new species were placed in the ‘Indochina – SW China’ clade (Shih et al. 2009) with strong support (Fig. 12). *Eosamon daiae* sp. nov. clusters with *E. tengchongense* and *E. lushuiense* and *Indochinamon malipoense* sp. nov. clusters with *I. tannanti* (Fig. 12).

## Discussion

The two new species cluster with several congeneric taxa (but not all), which tentatively supports recognition of the two genera, *Eosamon* and *Indochinamon*, following the systematic revision of Yeo and Ng (2007). However, based on our molecular analyses, *Eosamon* and *Indochinamon* are not monophyletic (Fig. 12). *Eosamon boonyaratae* (Naiyanetr, 1987), *E. smithianum* (Kemp, 1923) and *E. yotdomense* (Naiyanetr, 1984) were placed in the ‘Indochina’ clade instead of the ‘Indochina – SW China’ clade, suggesting a polyphyletic topological structure for the current composition of *Eosamon* sampled to date. Morphologically, some characters, e.g., carapace dorsally convex and male pleon with straight lateral margins, in *E. daiae* sp. nov., *E. tumidum*, *E. lushuiense* and *E. tengchongense*, distributed in China, also differ from the description of *Eosamon* that was proposed based on specimens of the species distributed in Thailand, Laos and Vietnam (Yeo and Ng 2007). Several relatives, *Potamiscus yiwuensis* Dai & Cai, 1998, *Pupamon nayung* (Naiyanetr, 1993) and *Beccumon jarujini* (Ng & Naiyanetr, 1993), are nested within the *Indochinamon* clade suggesting that *Indochinamon* is paraphyletic (Fig. 12). Ng and Mar (2018) separated *Indochinamon* into several groups on the basis of their G1 structures. Although only few *Indochinamon* species were included, our molecular results indicate that their classification is still problematic. *Indochinamon tannanti* (Rathbun, 1904) is genetically closer to *Beccumon* Yeo & Ng, 2007, and *Pupamon* Yeo & Ng, 2007, rather than *I. ou* (Yeo & Ng, 1998). Due to the lack of taxa and sampling of molecular markers, we could not delve deeper into these questions in the present study. Further studies are needed to clarify the systematic treatments of *Eosamon* and *Indochinamon*.

*Eosamon daiae* sp. nov. and *Indochinamon malipoense* sp. nov. are not threatened by human activity. *Eosamon daiae* sp. nov. is distributed in the vicinity of the Tongbiguan Nature Reserve and *Indochinamon malipoense* sp. nov. is distributed in the vicinity of the Laoshan Nature Reserve. In these areas, large-scale developments are strictly regulated.

Yunnan is a global biodiversity hotspot (Myers et al. 2000), and also an important center for global biodiversity and endemism of primary freshwater crabs (Cumberlidge et al. 2011). Generations of scientists have done plenty of species discovery of freshwater crabs in this area (reviewed by Dai 1999; Chu et al. 2018b). However, investigations of freshwater crabs on the Sino-Burmese border, Sino-Vietnamese border and Sino-Lao border have rarely been carried out, because of the proximity of the ‘Golden Triangle’. With constant efforts by the governments, conducting field surveys in these areas became possible. Many species have been newly described (e.g., Yu et al. 2019; Zhao et al. 2019; Lin and Li 2020; Zhang et al. 2020). In addition, some old type localities of freshwater crabs from Myanmar, e.g., *Indochinamon andersonianum* (Wood-Mason, 1871), *I. edwardsii* (Wood-Mason, 1871) and *I. hispidum* (Wood-Mason, 1871), are within Yunnan Province, China nowadays due to changes of national boundaries over one hundred years ago (Ng and Mar 2018). To fully understand the biodiversity of freshwater crabs in Yunnan, further investigations are expected in the poorly sampled frontier zones of China.

## Supplementary Material

XML Treatment for Eosamon daiae

XML Treatment for Indochinamon malipoense
